# Association between intensity of physical activity and cognitive function in hypertensive patients: a case–control study

**DOI:** 10.1038/s41598-024-59457-x

**Published:** 2024-05-02

**Authors:** Shunxin Lv, Huachen Jiao, Xia Zhong, Ying Qu, Mengdi Zhang, Rui Wang

**Affiliations:** 1 First Clinical Medical School, Shandong University of Traditional Chinese Medicine, No. 42, Wenhua West Road, Lixia District, Jinan, Shandong People’s Republic of China; 2 Department of Cardiology, Affiliated Hospital of Shandong University of Traditional Chinese Medicine, No. 42, Wenhua West Road, Lixia District, Jinan, Shandong People’s Republic of China; 3 Shandong University of Traditional Chinese Medicine, Jinan, People’s Republic of China

**Keywords:** Hypertension, Physical activity, Cognitive impairment, Cardiac structure, Neurological disorders, Cardiovascular biology

## Abstract

Previous studies have shown that a higher intensity of physical activity (PA) is associated with a lower risk of cognitive impairment (CI), whereas hypertension is associated with higher CI. However, there are few studies on the association between PA intensity and cognitive function in hypertensive patients. This study investigated the association between PA intensity and cognitive function in hypertensive patients. A total of 2035 hypertensive patients were included in this study, including 407 hypertensive patients with CI and 1628 hypertensive patients with normal cognitive function matched 1:4 by age and sex. The International Physical Activity Questionnaire-Long Form and the Mini-mental State Examination were used to evaluate PA intensity, total metabolic equivalents, and cognitive function in patients with hypertension. Multivariate logistic regression was used to analyze the correlation between PA intensity and CI in hypertensive patients. The Spearman correlation coefficient was used to analyze the correlation between PA intensity and the total score of each component of the MMSE and the correlation between PA total metabolic equivalents and cardiac structure in hypertensive patients. After adjusting for all confounding factors, PA intensity was negatively associated with CI in hypertensive patients (OR = 0.608, 95% CI: 0.447–0.776, P < 0.001), and this association was also observed in hypertensive patients with education level of primary school and below and junior high school and above (OR = 0.732, 95% CI: 0.539–0.995, P = 0.047; OR = 0.412, 95% CI: 0.272–0.626, P < 0.001). The intensity of PA in hypertensive patients was positively correlated with orientation (r = 0.125, P < 0.001), memory (r = 0.052, P = 0.020), attention and numeracy (r = 0.151, P < 0.001), recall ability (r = 0.110, P < 0.001), and language ability (r = 0.144, P < 0.001). PA total metabolic equivalents in hypertensive patients were negatively correlated with RVEDD and LAD (r = − 0.048, P = 0.030; r = − 0.051, P = 0.020) and uncorrelated with LVEDD (r = 0.026, P = 0.233). Higher PA intensity reduced the incidence of CI in hypertensive patients. Therefore, hypertensive patients were advised to moderate their PA according to their circumstances.

## Introduction

Elevated systolic blood pressure is one of the attributable risk factors for mortality worldwide^1^, and there were 540 million more people with hypertension in 2015 than in 1975, mainly in low- and middle-income countries^2^. Hypertension can not only increase the risk of cardiovascular disease, chronic kidney disease, stroke, and other diseases^3^ but also is a risk factor for cognitive impairment (CI)^4^. According to statistics, the prevalence of mild cognitive impairment (MCI) in hypertensive patients is 30%^5^. CI increases the economic burden of patients and affects their lives, which has become a public concern. Unfortunately, there are no specific drugs for the treatment of CI^6^. Therefore, it is particularly important to prevent and delay CI.

Physical activity (PA) is closely related to human health. Approximately 6–10% of the incidence of non-communicable diseases such as coronary atherosclerotic heart disease, type 2 diabetes, breast cancer, and colon cancer are caused by lack of PA, and enhanced PA is expected to add 0.68 years of life expectancy to the world population^7^. In addition, PA can not only control and prevent hypertension^8,9^, but also reduce the occurrence of CI^10^ and improve cardiac structure in hypertensive patients^11^. However, there are fewer studies on the correlation of PA with CI and cardiac structure in hypertensive patients.

This study aimed to investigate the relationship between PA intensity and cognitive function in hypertensive patients, as well as the correlation between total metabolic equivalents of PA and cardiac structure in hypertensive patients.

## Materials and methods

### Study design and data sources

All data involved in this case–control study were collected from outpatients or wards of 9 hospitals, including the Affiliated Hospital of Shandong University of Traditional Chinese Medicine, Jinan Hospital of Traditional Chinese Medicine, Jinan Minzu Hospital, Qingzhou People's Hospital, Penglai Hospital of Traditional Chinese Medicine, Tai 'an First People's Hospital, Guangrao County Hospital of Traditional Chinese Medicine, Qufu Hospital of Traditional Chinese Medicine, and Weifang Hospital of Traditional Chinese Medicine. These hypertensive patients were recorded in the hypertension electronic community follow-up system of the Affiliated Hospital of Shandong University of Traditional Chinese Medicine. A total of 4011 hypertensive cases of the system from May 2022 to October 2023 were exported and summarized into Excel tables. After excluding 26 cases with severe missing data among hypertension cases, the total number of included cases was 3985. Hypertension was diagnosed according to one of the following criteria: (1) office systolic blood pressure (SBP) ≥ 140 mmHg and/or diastolic blood pressure (DBP) ≥ 90 mmHg on 3 times on different days without using antihypertensive drugs. (2) If the patient has a history of hypertension and is currently using antihypertensive drugs, hypertension should still be diagnosed even if the blood pressure is < 140/90 mmHg. (3) The average SBP/DBP on 24-h ambulatory blood pressure monitoring ≥ 130/80 mmHg; daytime ≥ 135/85 mmHg; and nighttime ≥ 120/70 mmHg. (4) SBP/DBP ≥ 135/85 mmHg measured at home 3 times on different days without using antihypertensive drugs^12^. The Mini-mental State Examination (MMSE) assessed cognitive function. Normal cognitive function was defined as an MMSE score between 27 and 30, and CI was defined as an MMSE score < 27^13^. The inclusion criteria were as follows: (1) age between 40 and 79 years old; (2) patients with essential hypertension; (3) signing informed consent; and (4) voluntary completion of the collection of hypertension-related information. Exclusion criteria: (1) patients with various types of secondary hypertension; (2) patients with vascular dementia or cognitive decline due to vascular factors; (3) received new drug clinical trials within the past 3 months; (4) pregnant women, pre-pregnant women, and lactating women; (5) comorbid psychosis and/or psychotropic substance use disorder or dependence; (6) malignant tumors and severely impaired liver, kidney, and heart function. Patients with CI and normal cognitive function were matched by age and sex (1:4), and 2035 patients were enrolled, including 407 patients with CI and 1628 patients with normal cognitive function.

### Assessment of cognitive function

The Chinese version of the MMSE can effectively and reliably assess the cognitive function of the Chinese population^14^. The MMSE covers seven simple task domains: time and place, repeated words, arithmetic, language, and motor skills, divided into five sections: orientation (10 points), memory (3 points), attention and calculation (5 points), recall (3 points), and language (9 points), on a scale of 0 to 30, with higher scores indicating better cognitive function^15^.

### Assessment of physical activity

The International Physical Activity Questionnaire-Long Form (IPAQ-L) can validly and reliably assess the intensity of PA in the Chinese population^16,17^. The IPAQ-L is used to assess daily activities of work, daily living, daily transportation, and physical and recreational activities over the past 7 days. The IPAQ-L data are converted into metabolic equivalent task scores (METs) for each domain or intensity of PA. PA per week (MET-min/week) is calculated by multiplying the total number of minutes per category of activity by the specific METs for that activity and then adding the MET for each category. There are 3.3 METs for walking, 4 METs for moderate activity, 6 METs for cycling, and 8 METs for vigorous activity. PA is classified into three categories: (1) Low intensity (category 1): This is the lowest level of PA. Patients do not meet the criteria for category 2 or 3. (2) Moderate intensity (category 2): any one of the following three criteria: (1) vigorous activity for at least 20 min per day for more than 3 days; (2) Do at least 30 min of moderate intensity activity or walking daily for more than 5 days; (3) 5 or more days of any combination of walking, moderate or vigorous intensity activity to achieve a minimum of at least 600 MET-min/week. (4) High intensity (category 3): any one of the following two criteria: (1) Vigorous activity for at least 3 days with a cumulative of at least 1500 MET-min/week; (2) at least 3000 MET-min/week of any combination of 7 days of walking at moderate- or high-intensity^18^.

### Measurement of cardiac structure

Left ventricular end-diastolic diameter (LVEDD), right ventricular end-diastolic diameter (RVEDD), and left atrial diameter (LAD) were measured by a professional cardiac color Doppler technician in the hospital using the American GE color Doppler ultrasound instrument. LVEDD and RVEDD were measured at the level of the mitral tendon cable in parasternal left ventricular long-axis view at end-diastole. At the end of ventricular systole, LAD was measured by taking a vertical line from the posterior wall of the distal aorta to the posterior wall of the left atrium from the parasternal long axis view of the left ventricle, which was also the anteroposterior diameter of the left ventricle^19^.

### Assessment of sleep quality

The Pittsburgh Sleep Quality Index (PSQI) is a widely used sleep quality assessment questionnaire with good reliability and validity. The scale consists of 19 items, including 7 elements :(1) subjective sleep quality; (2) sleep latency; (3) sleep duration; (4) sleep efficiency; (5) sleep disorders; (6) sleeping pills; and (7) daytime dysfunction. Each component is scored on a scale of 0 to 3, and the sum of the seven component scores yields a subjective sleep quality score (range 0 to 21 points). The higher the score, the worse the subjective sleep quality^20^.

### Indicators of screening

We screened baseline data from all patients with hypertension, including basic information: age, gender (male/female), education level (primary school and below/junior high school and above), type of work (physical work group, mental work mainly, and both), smoking (no/yes), alcohol drinking (no/yes), course of hypertension, classification of hypertension (grade 1, 2 and 3), systolic pressure at ordinary times, diastolic pressure at ordinary times, waist circumference (WC), hip circumference (HC), body mass index (BMI): weight/height^^2^, Concomitant medications (no/yes): calcium channel blocker (CCB), diuretic, angiotensin-converting enzyme inhibitor or angiotensin-receptor inhibitor (ACEI or ARB), β-blocker, sympathetic nerve inhibitor, auxiliary examination: serum metabolic indexes (total cholesterol (TC), triglyceride (TG), low-density lipoprotein cholesterol (LDL-C), high-density lipoprotein cholesterol (HDL-C), fasting blood glucose (FBG), serum creatinine (Scr)), LVEDD, RVEDD, LAD, and subjective evaluation scales: IPAQ-L, PSQI, MMSE.

## Statistical analysis

All statistical analyses were performed using SPSS software (version 26.0; SPSS Inc., Chicago, IL, USA) and GraphPad Prism software (version 9.0.0; GraphPad Software, San Diego, CA, USA). Quantitative data were expressed as mean ± standard deviation or median and interquartile range and compared by *t*-test analysis or Mann–Whitney *U* test. Qualitative data were expressed as percentages and compared using the Chi-square test. Meanwhile, Pearson’s correlation test or Spearman’s correlation coefficient test was used to investigate the interrelationships. Multivariate logistic regression analysis was used to adjust for covariates. A two-tailed P-value < 0.05 was considered statistically significant.

### Ethics statement

This study followed the principles of the Declaration of Helsinki and was approved by the Ethics Committee of the Affiliated Hospital of Shandong University of Traditional Chinese Medicine ((2023) Review No. (109)-KY). Patients in the hypertension electronic community follow-up system signed written informed consent.

## Results

### Baseline characteristics of hypertensive patients

As shown in Table 1, 407 patients with CI (male/female: 189/218) and 1628 patients with normal cognition (male/female: 756/872) were enrolled. Compared with the control group, the CI group of hypertensive patients had greater age, WC, HC, and PSQI total score (P < 0.05), lower systolic pressure at ordinary times, serum TG, TC, and LDL-C levels, and PA total metabolic equivalent (P < 0.05), and higher proportion of education level of primary school and above, physical work as the type of work, no drinking, no CCB use, and low-intensity PA (P < 0.01).

**Table 1:** Baseline characteristics of hypertensive patients.

Variable	CI group (N = 407)	Control group (N = 1628)	P value
Basic information			
Age, year	70.71 ± 6.32	68.06 ± 6.46	< 0.001*
Gender, n (%)	189 (46.44)	756 (46.44)	1.000
Education level, n (%)	274 (67.32)	744 (45.70)	< 0.001*
Type of work, n (%)	330 (81.08)	953 (58.54)	< 0.001*
Smoking, n (%)	366 (89.93)	1436 (88.21)	0.330
Alcohol drinking, n (%)	373 (91.65)	1404 (86.24)	0.003*
Course of hypertension, month	100.00 (35.00, 172.00)	96.00 (37.00, 152.75)	0.425
Classification of hypertension, n (%)	46 (11.30)	193 (11.87)	0.496
Systolic pressure at ordinary times, mmHg	139.60 ± 13.48	142.03 ± 12.75	0.001*
Diastolic pressure at ordinary times, mmHg	84.71 ± 8.07	84.51 ± 9.50	0.664
WC, cm	89.01 ± 9.02	87.70 ± 10.46	0.011*
HC, cm	99.19 ± 10.80	97.12 ± 10.49	< 0.001*
BMI, kg/m^2^	25.18 ± 3.17	25.45 ± 3.22	0.120
Auxiliary examination			
FBG, mmol/L	5.90 (5.14, 7.20)	5.92 (5.22, 7.41)	0.186
TG, mmol/L	1.26 (0.89, 1.80)	1.35 (0.98, 1.88)	0.023*
TC, mmol/L	4.39 ± 1.15	4.61 ± 1.20	0.001*
HDL-C, mmol/L	1.24 ± 0.39	1.26 ± 0.38	0.281
LDL-C, mmol/L	2.53 ± 0.93	2.78 ± 0.98	< 0.001*
Scr, μmol/L	64.00 (53.00, 75.30)	63.30 (54.00, 76.30)	0.492
LVEDD, mm	47.00 ± 6.20	46.56 ± 5.64	0.167
RVEDD, mm	21.52 ± 4.14	21.58 ± 3.76	0.483
LAD, mm	36.12 ± 7.18	35.38 ± 5.66	0.051
Concomitant medications			
CCB, n (%)	234 (57.49)	756 (46.44)	< 0.001*
Diuretics, n (%)	339 (83.29)	1365 (83.85)	0.787
ACEI or ARB, n (%)	206 (50.61)	748 (45.95)	0.091
Beta-blockers, n (%)	322 (79.12)	1246 (76.54)	0.268
Sympathetic nerve inhibitors, *n* (%)	397 (97.54)	1602 (98.40)	0.239
Subjective assessment scale			
PA intensity, n (%)	56 (13.76)	135 (8.29)	< 0.001*
Total metabolic equivalents of PA, MET	1170.00 (724.00, 1980.00)	1622.00 (930.00, 2737.00)	< 0.001*
PSQI total score	8.00 (7.00, 10.00)	6.00 (4.00, 8.00)	< 0.001*

Data were presented as mean ± SD, median (25th, 75th) or n (%).*Statistically significant value (P < 0.05). *CI* cognitive impairment, *WC* waist circumference, *HC* hip circumference, *BMI* body mass index, *FBG* fasting blood glucose, *TG* triglyceride, *TC* total cholesterol, *HDL-C* high-density lipoprotein cholesterol, *LDL-C* low-density lipoprotein cholesterol, *Scr* serum creatinine, *LVEDD* left ventricular end-diastolic diameter, *RVEDD* right ventricular end-diastolic diameter, *LAD* left atrium diameter, *CCB* calcium channel blocker, *ACEI or ARB* angiotensin-converting enzyme inhibitor or angiotensin receptor inhibitor, *PA* physical activity, *PSQI* Pittsburgh Sleep Quality Index.

**Figure 1:**
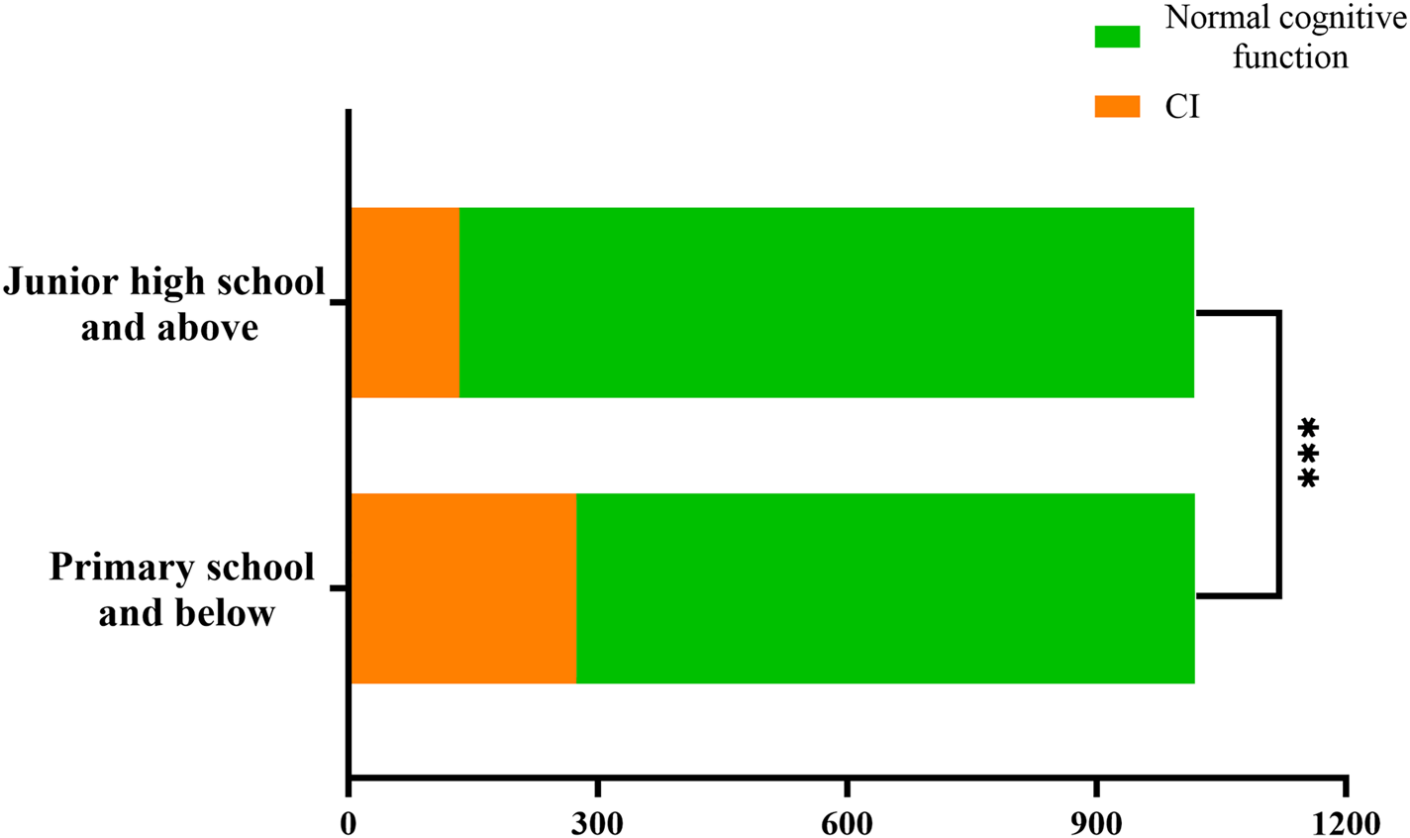
Differences in cognitive function among hypertensive patients with different educational levels. Compared with hypertensive patients with junior high school education and above, hypertensive patients with primary school education and below had a higher proportion of CI (26.92% vs 13.08%, P < 0.001). Statistically significant value (P < 0.001); *CI* cognitive impairment.

Figure 1 analyses of differences in cognitive function among hypertensive patients with different educational levels using the chi-square test. Compared with hypertensive patients with junior high school education and above, hypertensive patients with primary school education and below had a higher proportion of CI (26.92% vs 13.08%, P < 0.001).

### Relationship between PA intensity and cognitive function in hypertensive patients

Table 2 uses multivariate logistic regression to explore the relationship between PA intensity and cognitive function in hypertensive patients. After adjusting for age, type of work, alcohol drinking, CCB, ACEI or ARB, and PSQI total score, PA intensity was negatively associated with CI in hypertensive patients (OR = 0.594, 95% CI: 0.467–0.755, P < 0.001), and the negative relationship still existed in hypertensive patients with education level of primary school and below and junior high school and above (OR = 0.732, 95% CI: 0.541–0.990, P = 0.043; OR = 0.410, 95% CI: 0.275–0.612, P < 0.001). After adjusting for systolic pressure at ordinary times, WC, HC, serum TG, TC, and LDL-C levels, PA intensity was also negatively associated with CI in hypertensive patients (OR = 0.518, 95% CI: 0.415–0.645, P < 0.001), and the relationship was negative in hypertensive patients with education level of primary school and below and junior high school and above (OR = 0.636, 95% CI: 0.479–0.844, P = 0.002; OR = 0.373, 95% CI: 0.255–0.545, P < 0.001). After adjusting for all the above factors, PA intensity was still negatively associated with CI in hypertensive patients (OR = 0.608, 95% CI: 0.447–0.776, P < 0.001), and PA intensity was also negatively correlated with CI in hypertensive patients with education level of primary school and below and junior high school and above (OR = 0.732, 95% CI: 0.539–0.995, P = 0.047; OR = 0.412, 95% CI: 0.272–0.626, P < 0.001).

**Table 2:** The relationship between PA intensity and cognitive function in hypertensive patients.

	CI					
	Total		Primary school and below		Junior high school and above	
	OR (95% CI)	P value	OR (95% CI)	P value	OR (95% CI)	P value
Model 1	0.518 (0.417, 0.643)	< 0.001*	0.628 (0.475, 0.829)	0.001*	0.397 (0.277, 0.569)	< 0.001*
Model 2	0.594 (0.467, 0.755)	< 0.001*	0.732 (0.541, 0.990)	0.043*	0.410 (0.275, 0.612)	< 0.001*
Model 3	0.518 (0.415, 0.645)	< 0.001*	0.636 (0.479, 0.844)	0.002*	0.373 (0.255, 0.545)	< 0.001*
Model 4	0.608 (0.447, 0.776)	< 0.001*	0.732 (0.539, 0.995)	0.047*	0.412 (0.272, 0.626)	< 0.001*

Model 1: crude, no adjustment.

Model 2: adjusting for age, type of work, alcohol drinking, CCB, ACEI or ARB, and PSQI total score.

Model 3: adjusting for systolic pressure at ordinary times, WC, HC, serum TG, TC, and LDL-C levels.

Model 4: adjusting for all the above factors.

*Statistically significant value (P < 0.05)

*PA* physical activity, *CI* cognitive impairment, *CCB* calcium channel blocker, *ACEI or ARB* angiotensin-converting enzyme inhibitor or angiotensin-receptor inhibitor, *PSQI* Pittsburgh Sleep Quality Index, *WC* waist circumference, *HC* hip circumference, *TC* total cholesterol, *TG* triglyceride, *LDL-C* low-density lipoprotein cholesterol.

### Correlation between PA intensity and MMSE scores in hypertensive patients

Table 3 uses Spearman’s correlation coefficient to analyze the correlation between the intensity of PA and MMSE scores in hypertensive patients. The results showed that PA intensity in hypertensive patients was positively correlated with orientation (r = 0.125, P < 0.001), memory (r = 0.052, P = 0.020), attention and numeracy (r = 0.151, P < 0.001), recall ability (r = 0.110, P < 0.001), and language ability (r = 0.144, P < 0.001).

**Table 3:** Correlation PA intensity and each part of MMSE scores in hypertensive patients.

	PA types	
	r value	P value
Orientation	0.125	< 0.001*
Memory	0.052	0.020*
Attention and numeracy	0.151	< 0.001*
Recall ability	0.110	< 0.001*
Language ability	0.144	< 0.001*

*Statistically significant value (P < 0.05). *PA* physical activity, *MMSE* Mini-mental State Examination.

### Correlation between PA total metabolic equivalent and cardiac structure in hypertensive patients

**Figure 2:**
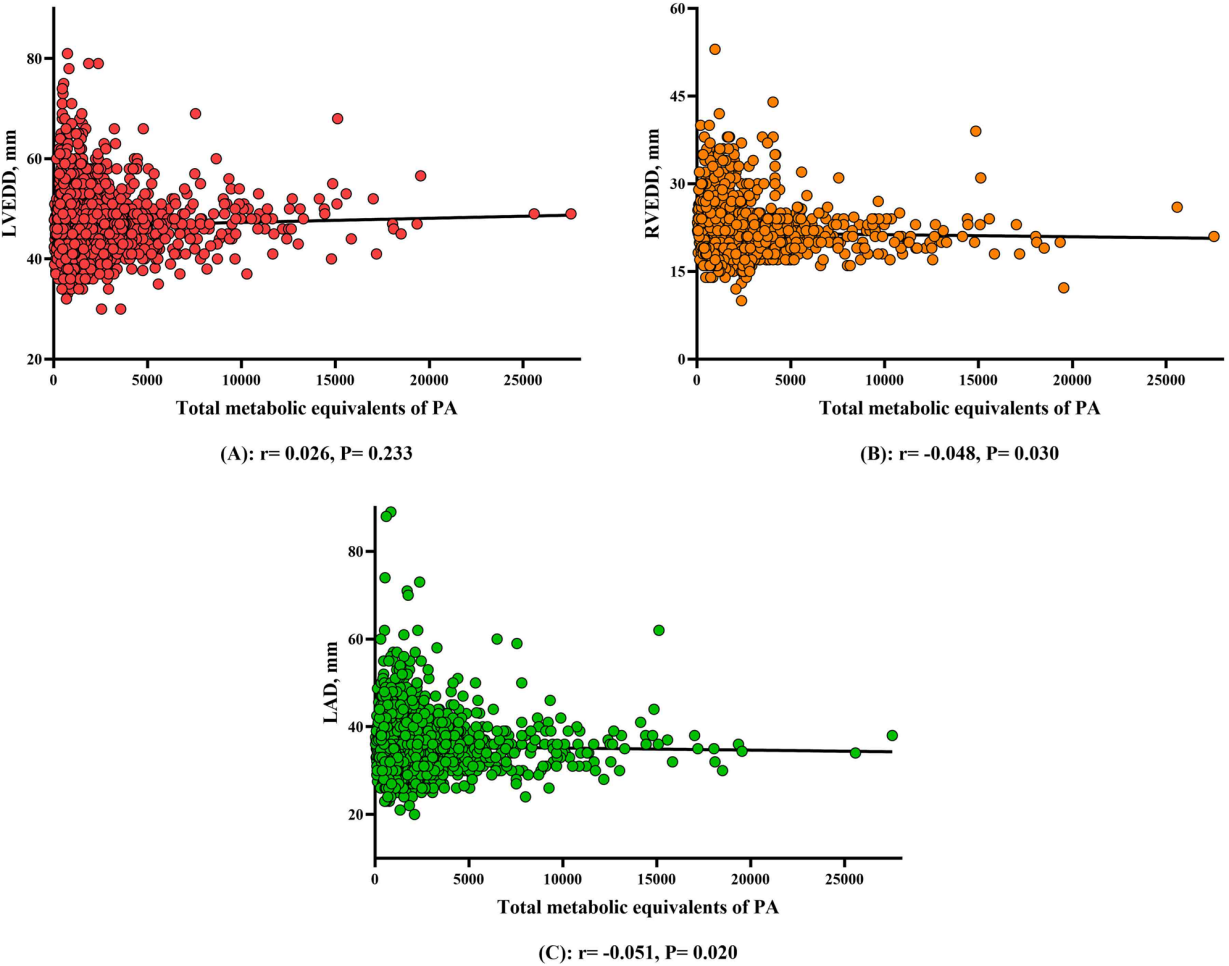
Correlation between PA total metabolic equivalent and cardiac structure in hypertensive patients. The total metabolic equivalent of PA was negatively correlated with RVEDD and LAD in hypertensive patients (r = − 0.048, P = 0.030 (**B**); r = − 0.051, P = 0.020 (**C**)). There was no correlation between PA total metabolic equivalent and LVEDD in hypertensive patients (r = 0.026, P = 0.233 (**A**)). *PA* physical activity, *RVEDD* right ventricular end-diastolic diameter, *LAD* left atrial diameter.

Figure 2 uses Spearman’s correlation coefficient to analyze the correlation between PA total metabolic equivalent and cardiac structure in hypertensive patients. The total metabolic equivalent of PA was negatively correlated with RVEDD and LAD in hypertensive patients (r = − 0.048, P = 0.030, Fig. 2B; r = − 0.051, P = 0.020, Fig. 2C). There was no correlation between PA total metabolic equivalent and LVEDD in hypertensive patients (r = 0.026, P = 0.233, Fig. 2A).

## Discussion

The purpose of this study was to investigate the association between PA intensity and cognitive function in hypertensive patients. The results showed that a higher PA intensity was associated with a lower incidence of CI in hypertensive patients, and this association persisted even after adjusting for all confounding factors. In addition, higher PA intensity still reduced the occurrence of CI in hypertensive patients with education levels of primary school and below and junior high school and above. In hypertensive patients, PA intensity was positively correlated with orientation, memory, attention and numeracy, recall ability, and language ability; the total metabolic equivalent of PA was negatively correlated with RVEDD and LAD and uncorrelated with LVEDD.

This study analyzed the differences in basic information, auxiliary examination, concomitant medications, and subjective evaluation scale between the CI group of hypertensive patients and the normal cognitive function group of hypertensive patients. The study found that hypertensive CI patients had greater age, WC, HC, and PA total metabolic equivalents, as well as poorer sleep quality, which was similar to the findings of several studies^21–24^. Among hypertensive patients, patients with CI were more likely to have an education level of primary school and above, do manual work, do not drink alcohol, and do not use CCB compared with those with normal cognitive function. The study found that the proportion of hypertensive patients with early CI who had an education level of primary school and below and were mainly engaged in physical work was higher than that of hypertensive patients with normal cognitive function^25^. A Mendelian randomization study found that older men who were infrequent drinkers and abstained from alcohol had greater odds of CI than those who were regular drinkers^26^. Animal experiments have shown that nifedipine not only increased cerebral blood flow in KK-Ay mice but also reduced high serum insulin levels and the production of superoxide and neural differentiation inhibitor protein Id-1 in KK-Ay mice^27^. This study also found that hypertensive patients with CI have lower systolic pressure at ordinary times and serum TG, TC, and LDL-C levels than hypertensive patients with normal cognitive function, which was inconsistent with the results of mainstream studies but similar to the results of some studies. A longitudinal cohort study showed a U-shaped association between systolic blood pressure and CI in older Chinese adults^28^. A cross-sectional study found that plasma TC, TG, and LDL-C levels were lower in elderly Chinese patients with CI than in those with normal cognitive function, which might be related to the inhibition of neuronal cell growth and functional integrity by low cholesterol^29^.

PA is a modifiable factor. Although the relationship between PA and CI has been less studied clinically in hypertensive patients, there is no shortage of studies on them in other populations. Two cross-sectional studies found that a Korean population that did not achieve moderate to high PA was associated with cognitive decline^11^; a higher PA level decreased the incidence of MCI in the Chinese population aged ≥ 60 years^30^. Zhong Xia et al. found that PA could be used as a predictor of early CI in hypertensive patients by lasso regression, and the lower the intensity of PA, the higher the risk of early CI in hypertensive patients^25^. Two meta-analyses and systematic reviews found that PA helped reduce the risk of cognitive decline^31,32^. Another systematic review showed that moderate-to-high PA was beneficial for improving cognitive function in different age groups, including learning behavior in children aged 6–13 and 14–18 years, and executive function, global cognition, and attention in people older than 50 years^33^. In addition, systematic review studies from the Chinese elderly population have shown that elevated PA levels reduce the incidence of MCI in the Chinese elderly^34^. This study showed that a higher PA intensity could reduce the occurrence of CI in hypertensive patients, and this relationship also existed in hypertensive patients with primary school and above and junior high school and above, which were similar to the results of the above studies. This study also found that stronger PA intensity was positively correlated with the total score of orientation, memory, attention and numeracy, recall ability, and language ability in hypertensive patients, which was similar to the conclusion of previous studies. Two cross-sectional studies separately found that ≥ 150 min of moderate-to-high-intensity PA reduced the prevalence of limitations in orientation and language ability in older Brazilian adults^35^; moderate-to-high-intensity PA also improved attention and language scores in older adults in northern China^30^. A Chinese longitudinal study found that higher PA levels helped delay the decline of episodic memory function in Chinese patients with diabetes^36^. In conclusion, a stronger intensity of PA can help reduce the occurrence of CI in hypertensive patients and improve cognitive domains such as orientation, memory, attention and numeracy, recall ability, and language ability in hypertensive patients.

The mechanism by which stronger PA can reduce the occurrence of CI in hypertensive patients may be related to improving cerebral blood supply and perfusion and promoting the growth of central nervous cells. Brain cells maintain normal growth and function by obtaining oxygen, glucose, and other essential nutrients through the cerebral circulation while excluding carbon dioxide, lactate, and other metabolites^37^. Decreased cardiac output and neuronal nitric oxide synthase levels were associated with reduced cerebral blood flow^38,39^, and total cerebral blood flow per 100 ml of brain parenchymal volume was positively correlated with cognitive function^40^. PA increased cardiac output^41^, and exercise increased neuronal nitric oxide synthase levels^42^, both of which improved cerebral blood flow, thereby preventing and delaying cognitive decline. As pro-inflammatory factors, tumor necrosis factor-α (TNF-α) and C-reactive protein (CRP) can damage central neuronal cells^43,44^ and are associated with the decline of cognitive function^45^; brain-derived neurotrophic factor (BDNF) and insulin-like growth factor (IGF-1) can protect and promote the development of central neurons^46,47^ and improve the cognitive function of patients^48,49^. A meta-analysis found that PA could reduce the levels of TNF-α and CRP, and promote the production of BDNF and IGF-1 in patients with MCI^50^. Hypertension itself could lead to the damage of brain neurons by aggravating oxidative stress in the brain, destroying brain microvessels and the blood–brain barrier, and damaging brain white matter and neurovascular coupling, thereby increasing the risk of CI^51^. Systematic reviews have found that PA can help hypertensive patients better control their blood pressure^52^. Therefore, PA may be more effective in reducing the risk of CI in hypertensive patients.

This study also found that the proportion of CI in hypertensive patients with primary school education and below was higher than that in hypertensive patients with junior high school education and above. Two cross-sectional studies found that Chinese populations with education levels of junior high school and above and primary school reduced the incidence of CI compared with those who were illiterate^53^; the proportion of illiteracy and primary school education in Chinese elderly with CI was higher compared with those with normal cognitive function^13^. It may be that a higher level of education is associated with greater cognitive reserve, thereby improving the cognitive function of patients^54^. In addition, this study found that PA total metabolic equivalents were negatively correlated with LAD in hypertensive patients, which was different from the results of previous studies. A prospective observational survey found that PA score was positively associated with LAD at 5 and 20 years of follow-up in a US population^55^; a case–control study found that PA level was positively associated with LAD in the Chinese population^56^. Differences in the study populations and PA variables may have contributed to the findings of this study being different from those of the other two studies. This study also found that PA total metabolic equivalents were negatively correlated with RVEDD in hypertensive patients, which has rarely been reported in previous studies.

There are several limitations to this study. First, this study was a case–control study, and it was not possible to determine the causal relationship between PA intensity and CI in hypertensive patients. Therefore, future prospective cohort studies are warranted to determine this relationship. Second, although the multivariate logistic regression model was used to correct confounders in this study, the possibility of residual bias caused by other relevant confounders not included in this study cannot be ruled out. Finally, the samples included in this study were limited to patients with hypertension who visited outpatient clinics or wards of hospitals in Shandong Province, China, and future studies with large samples and multiple regions are needed. However, this study found that higher PA intensity reduced the occurrence of CI in hypertensive patients, and this relationship also existed in hypertensive patients with education levels of primary school and below and junior school and above. Therefore, it recommends hypertensive patients moderately PA according to their conditions.

## Conclusion

This study showed that a higher PA intensity reduced the occurrence of CI in hypertensive patients, and this association was also present in hypertensive patients with education levels of primary school and below and junior high school and above. This mechanism may be related to improving brain blood supply and perfusion and promoting the growth of central nervous cells. Therefore, it recommends hypertensive patients moderately PA according to their conditions.

## Data Availability

The raw data supporting the conclusions of this article will be made available by the corresponding author, without undue reservation.

## Abbreviations

CI: Cognitive impairment
MCI: Mild cognitive impairment
PA: Physical activity
MMSE: Mini-mental State Examination
IPAQ-L: International Physical Activity Questionnaire-Long Form
METs: Metabolic equivalent task scores
LVEDD: Left ventricular end-diastolic diameter
RVEDD: Right ventricular end-diastolic diameter
LAD: Left atrial diameter
PSQI: Pittsburgh Sleep Quality Index
WC: Waist circumference
HC: Hip circumference
BMI: Body mass index
CCB: Calcium channel blocker
ACEI or ARB: Angiotensin-converting enzyme inhibitor or angiotensin-receptor inhibitor
TC: Total cholesterol
TG: Triglyceride
LDL-C: Low-density lipoprotein cholesterol
HDL-C: High-density lipoprotein cholesterol
FBG: Fasting blood glucose
Scr: Serum creatinine
TNF-α: Tumor necrosis factor-α
CRP: C-reactive protein
BDNF: Brain-derived neurotrophic factor
IGF-1: Insulin-like growth factor
